# Perivascular space fluid diffusivity predicts clinical deterioration in prodromal and early-stage Parkinson’s disease

**DOI:** 10.1038/s41531-025-01036-6

**Published:** 2025-06-14

**Authors:** Yi Xing, Miao Lin, Jingzhe Li, Xiaoran Huang, Lei Yan, Jingru Ren, Hao Zhou, Shuoying Chen, Yiting Cao, Peiyu Huang, Weiguo Liu

**Affiliations:** 1 Department of Neurology, The Affiliated Brain Hospital of Nanjing Medical University, Nanjing, China; 2 Department of Radiology, The Second Affiliated Hospital, Zhejiang University School of Medicine, Hangzhou, China; 3 Department of Radiology, The First Affiliated Hospital of Wenzhou Medical University, Wenzhou, China

**Keywords:** Parkinson's disease, Diffusion tensor imaging, Neurological disorders

## Abstract

The glymphatic system is essential for clearing toxic proteins from the brain, and understanding its dysfunction in the early stages of Parkinson’s disease (PD) may facilitate the development of disease-modifying therapies. This study aimed to evaluate alterations in glymphatic function and its correlation with disease progression in prodromal and early clinical stages of PD. Participants were categorized into three groups: prodromal PD (pPD), de novo PD (dnPD), and healthy controls (HCs), further divided by age. Glymphatic function was assessed using the ALPS index derived from diffusion tensor imaging. Results indicated that the ALPS index was significantly lower in older pPD and dnPD patients, correlating with various clinical symptoms. Longitudinal analysis revealed a decrease in the ALPS index over time in pPD patients who progressed to clinical PD, while it remained stable in non-converters. Additionally, the baseline ALPS index was predictive of the progression of both motor and non-motor symptoms in pPD patients. In dnPD patients, a lower baseline ALPS index predicted the progression of motor symptoms in the older subgroup. Overall, the ALPS index is reduced in the early stages of PD and may serve as a predictor for disease progression.

## Introduction

The onset and progression of Parkinson’s disease (PD) are insidious, characterized by irreversible degeneration and death of neurons^1^. Therefore, early intervention is crucial. Nevertheless, current treatments, such as dopamine replacement therapy, fail to modify the progression of the disease^2^. It is imperative to elucidate additional mechanisms contributing to the pathogenesis and progression of PD and to develop novel disease-modifying treatment strategies for early intervention.

The characteristic pathological protein, α-synuclein, can be released into extracellular space and subsequently internalized by other cells, causing ‘Prion-like’ propagation^3^. The glymphatic system facilitates the clearance of various misfolded proteins in the extracellular space, including α-synuclein^3,4^. Its impairment has thus been implicated in the pathogenesis and progression of PD^5^. Understanding the role of this clearance system in the prodromal and early clinical stages of PD and its association with disease progression could unveil potential therapeutic targets.

Diffusion tensor image analysis along the perivascular space (DTI-ALPS) is a noninvasive method for evaluating glymphatic function^6^, and its association with glymphatic clearance has been validated by dynamic contrast enhanced magnetic resonance imaging (DCE-MRI)^7^. While the method has been applied in several studies in PD, it remains controversial whether the ALPS index declined at early stages^8–12^. Two studies found decreased ALPS index^11,12^, but the others did not^8–10^. Longitudinal studies examining the correlation between baseline ALPS index and the progression of motor symptoms in PD remain limited, and the results were also inconsistent^11,13^. Interestingly, two studies reported a decrease in the ALPS index in patients with rapid eye movement sleep behavior disorder (RBD)^11,14^, indicating a potentially much earlier involvement of the glymphatic dysfunction. However, studies involving RBD patients do not fully represent pPD, and the relationship between the ALPS index and the conversion from prodromal to clinically diagnosed PD remains unclear. Therefore, further studies in well-defined longitudinal cohorts are needed to understand how glymphatic function changes and impacts disease progression during the prodromal and early clinical stages of PD.

In addition, previous research has consistently reported a negative correlation between age and the ALPS index in PD patients^8,9,15^, underscoring the critical role of aging in glymphatic function. Notably, a study reported a significant decline in the ALPS index after the age of 65^16^. Despite these findings, impairments in glymphatic function and their contributions to disease progression across different age groups in PD remains unknown. Clarifying this issue will facilitate a more precise understanding of how aging modulates the association between glymphatic function and PD.

Given the identified gaps and limitations in previous research, our study aimed to: (1) investigate how glymphatic function changes during the prodromal and early clinical stages of PD across different age groups; (2) investigate the impact of glymphatic dysfunction on the conversion from prodromal to clinically diagnosed PD and the deterioration of clinical symptoms. Through this study, we hoped to provide new insights into the mechanisms of PD pathogenesis and progression, thereby contributing to the development of novel disease-modifying therapies.

## Results

### Demographics and clinical data

Demographic and clinical information are summarized in Table 1. Totally, 89 HCs, 91 pPD patients, and 183 dnPD patients were included. There were no significant differences among the groups in terms of sex and age (p > 0.05). However, participants in the dnPD group had a lower level of education (9.20 ± 4.47) compared to those in the HC group (10.81 ± 3.91). The UPDRS-III score was higher in the dnPD group (24.11 ± 8.20) than in the pPD group (8.68 ± 4.17). Regarding cognitive performance, individuals in the HC group (26.79 ± 1.47) exhibited higher MoCA scores compared to those in the pPD (22.81 ± 4.51) and dnPD (21.49 ± 5.26) groups. No significant inter-group differences were observed for PDNMS, HAMA, and HAMD (p > 0.05). After further subgrouping the subjects based on whether their age was ≥ 65 years, there remained no statistically significant differences in age or sex among the three subgroups (p > 0.05). Among participants younger than 65 years, the dnPDm group had a lower level of education (9.68 ± 4.24 years) compared to the HCm (10.82 ± 3.76 years) and pPDm groups (10.96 ± 4.22 years). Detailed data are presented in Tables S2, S3.

**Table 1:** Demographics and Clinical Data

	HC (n = 89)	pPD (n = 91)	dnPD (n = 183)		Post-hoc		
				P	P*	P**	P***
Age (years)	57.97 ± 5.29	60.18 ± 9.09	59.43 ± 8.34	0.160^d^	-	-	-
Sex (Male %)	47.19	52.74	49.18	0.748^b^	-	-	-
Disease duration (years)	-	-	1.97 ± 2.36	**-**	-	**-**	-
Education (years)	10.81 ± 3.91	10.34 ± 4.32	9.20 ± 4.47	**0.002**^c^	1.000	**0.012**	0.163
H-Y stage	-	-	2(1.5, 2)	**-**	-	-	-
UPDRS-II	-	-	7.76 ± 3.67	**-**	-	-	-
UPDRS-III	-	8.68 ± 4.17	24.11 ± 8.20	**<0.001**^d^	-	-	-
PDSS	-	-	125.55 ± 24.07	-	-	-	-
PDNMS	-	8.42 ± 4.92	7.69 ± 4.33	0.282^d^	-		-
MoCA	26.79 ± 1.47	22.81 ± 4.51	21.49 ± 5.26	**<0.001**^c^	**<0.001**	**<0.001**	0.065
HAMA	-	6.73 ± 4.98	6.70 ± 5.41	0.804^d^	**-**	**-**	-
HAMD	-	8.12 ± 5.89	9.38 ± 6.84	0.204^d^	**-**	**-**	-

*HC* healthy control, *pPD* prodromal Parkinson’s disease, *dnPD* de novo Parkinson’s disease, *H-Y* Hoehn and Yahr, *UPDRS-II* Unified Parkinson’s Disease Rating Scale, Part II, *UPDRS-III* Unified Parkinson’s Disease Rating Scale, Part III, *PDSS* Parkinson’s Disease Sleep Scale, *PDNMS* Parkinson’s disease Non-Motor Symptoms Questionnaire, *MoCA* Montreal Cognitive Assessment, *HAMD* Hamilton Depression Rating Scale, *HAMA* Hamilton Anxiety Scale.

P*: HC vs pPD, P**: HC vs dnPD, P***: pPD vs dnPD.

^a^Analysis of Variance.

^b^chi-square tests.

^c^Kruskal–Wallis W test.

^d^Mann-Whitney U test.

Bold values indicate statistical significance (P < 0.05).

**Fig. 1:**
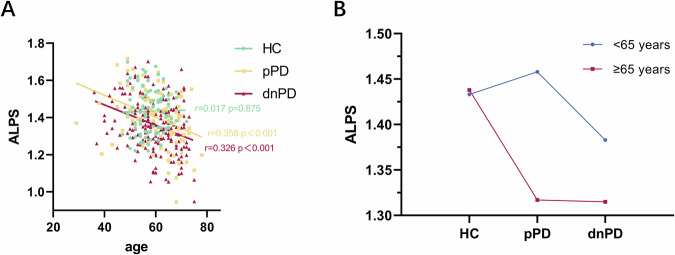
Association of the ALPS index with age and diagnostic status. **A** Correlations of the ALPS index and age in the 3 groups. **B** The changing trend of the ALPS index in three groups of patients in different age groups. Significant main effects were observed for age subgroup (F = 15.743, p < 0.001) and diagnostic status (F = 8.453, p < 0.001) on the ALPS index. Additionally, a significant interaction effect between age subgroup and diagnostic status was found (F = 5.081, p = 0.007).

The ALPS index was found to be negatively correlated with age in pPD and dnPD cohorts (Fig. 1A). Moreover, significant main effects were observed for age subgroup (F = 15.743, p < 0.001) and diagnostic status (F = 8.453, p < 0.001) on the ALPS index. A significant interaction effect between age subgroup and diagnostic status was also found (F = 5.081, p = 0.007), as depicted in Fig. 1B. Therefore, age was considered and controlled as a significant confounder in subsequent analyses.

### Cross-sectional results

**Fig. 2:**
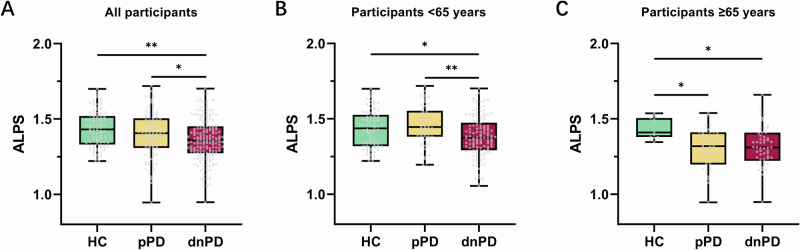
Results of inter-group comparison of the ALPS index. **A** The ALPS index among the 3 groups. **B** The ALPS index among the 3 subgroups aged less than 65 years old. **C** The ALPS index among the 3 subgroups aged 65 years or older. ANCOVA was used to examine group differences, controlling for age, sex, and education level. *P < 0.05, **P < 0.01, and ***P < 0.001. ALPS, diffusion tensor image analysis along the perivascular space; HC healthy controls, dnPD de novo Parkinson’s disease, pPD prodromal Parkinson’s disease.

As depicted in Table S1 and Fig. 2, dnPD group exhibited a significantly lower ALPS index (1.359 ± 0.140) compared to pPD (1.396 ± 0.152) and HC groups (1.434 ± 0.119). This difference was consistent among participants aged less than 65 years old, with the ALPS index being lower in the dnPDm group (1.381 ± 0.133) compared to pPDm (1.458 ± 0.129) and HCm group (1.433 ± 0.124). However, among participants aged 65 years or more, the difference in ALPS index between the pPDo (1.306 ± 0.139) and dnPDo (1.314 ± 0.143) groups was no longer significant. Instead, the ALPS index was significantly lower in the pPDo and dnPDo groups compared to the HCo group (1.438 ± 0.069).

In the pPD cohort (Table S4), there was no significant correlation between the ALPS index and clinical features in the entire group. The ALPS index exhibited a positive correlation with the MoCA score (r = 0.427, p = 0.002, FDR-p = 0.006) in the pPDm subgroup, and this association remained significant after further adjustment for free water values in the posterior substantia nigra (PSN-FW) as an additional covariate (r = 0.463, p = 0.001, FDR-p = 0.003; Table S5).

In the dnPD cohort (TABLE S4), a positive correlation was observed between PDSS score and the ALPS index (r = 0.184, p = 0.014, FDR-p = 0.048). Conversely, H-Y stage (r = −0.186, p = 0.012, FDR-p = 0.048) and PDNMS (r = −0.176, p = 0.018, FDR-p = 0.048) score were negatively associated with the ALPS index. Upon dividing participants into two subgroups based on age, the UPDRS-II (r = −0.309, p = 0.020, FDR-p = 0.032), UPDRS-III (r = −0.299, p = 0.025, FDR-p = 0.033), PDNMS (r = −0.369, p = 0.005, FDR-p = 0.013), PDSS (r = 0.433, p < 0.001, FDR-p = 0.007), HAMA (r = −0.314, p = 0.019, FDR-p = 0.032) and HAMD (r = −0.375, p = 0.004, FDR-p = 0.013) scores were significantly correlated with the ALPS index in the dnPDo subgroup. And no clinical features were correlated with the ALPS index in the dnPDm subgroup after multiple comparison correction. Similarly, to further investigate potential confounding effects of dopaminergic dysfunction, we further included PSN-FW as an additional covariate in our analyses and observed that the correlations between the ALPS index and clinical measures in both dnPDo and dnPDm subgroups remained largely unchanged (Table S5). However, in the entire dnPD cohort, when controlling for PSN-FW, the previously observed correlations between the ALPS index and H-Y stage, PDSS, and PDNMS no longer achieved statistical significance after FDR correction (FDR-p = 0.056; Table S5).

### Longitudinal results

**Fig. 3:**
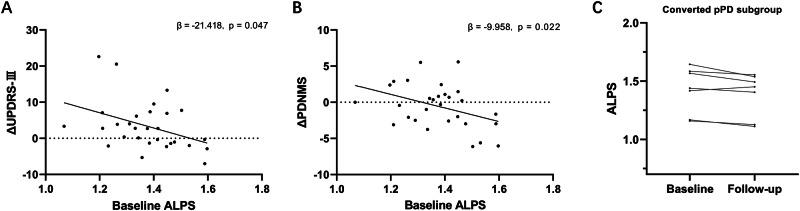
Longitudinal study of pPD cohort. **A** Correlations of the baseline ALPS index with ΔUPDRS-III score in entire pPD cohort. **B** Correlations of the baseline ALPS index with ΔPDNMS score in entire pPD cohort. **C** Longitudinal ALPS index in converted pPD subgroup. ΔUPDRS-III and ΔPDNMS are defined as the value at the end of follow-up minus the baseline value. Controlling for age, sex, education level, and baseline UPDRS-III or PDNMS scores.

Among all pPD participants, 29 completed longitudinal follow-up, with an average follow-up time of 1.7 years (1.73 ± 0.66 years). The age of participants who completed follow-up (64.86 ± 6.82 years) was significantly higher than that of those who did not (57.98 ± 9.24 years). No significant differences were observed in other demographic or clinical data between the groups (Table S6). Longitudinal changes in the clinical features and the ALPS index of these subjects are presented in Table 2. UPDRS-III (β = 1.602, SE = 0.724, t = 2.213, p = 0.035) and MoCA scores (β = 0.725, SE = 0.274, t = 2.642, p = 0.013) exhibited a significant increase over time. Although the ALPS index was not significantly decreased (β = −0.006, SE = 0.006, t = −0.974, p = 0.338), a lower baseline ALPS index was associated with a greater increase in UPDRS-III (β = −21.418, 95% CI: −42.570 to −0.266, p = 0.047; Fig. 3A, Table S8) and PDNMS scores (β = −9.958, 95% CI: −18.310 to −1.606, p = 0.022; Fig. 3B, Table S8), after controlling for age, sex, education level, and the corresponding baseline clinical features. Additionally, during the follow-up, 7 pPD participants converted to PD, among whom 6 exhibited a decline in the ALPS index (Fig. 3C), although the time effect was not significant in the LMM (β = −0.017, SE = 0.009, t = −1.930, p = 0.101).

**Table 2:** Time Effects in Linear Mixed-Effects Models for Longitudinal Changes in Clinical Features and the ALPS Index in the pPD Cohort

	β (SE)	t	P-value
UPDRS-III	1.602(0.724)	2.213	**0.035**
MoCA	0.725(0.274)	2.642	**0.013**
PDNMS	−0.268(0.340)	−0.789	0.436
ALPS	−0.006(0.006)	−0.974	0.338

Linear mixed-effects models included time, sex, and age as fixed effects.

*UPDRS-III* Unified Parkinson’s Disease Rating Scale, Part III, *MoCA* Montreal Cognitive Assessment, *PDNMS* Parkinson’s disease Non-Motor Symptoms Questionnaire, *ALPS* diffusion tensor image analysis along the perivascular space.

All participants included in our pPD cohort received a second MRI scan at the end of follow-up.

Bold values indicate statistical significance (P < 0.05).

**Fig. 4:**
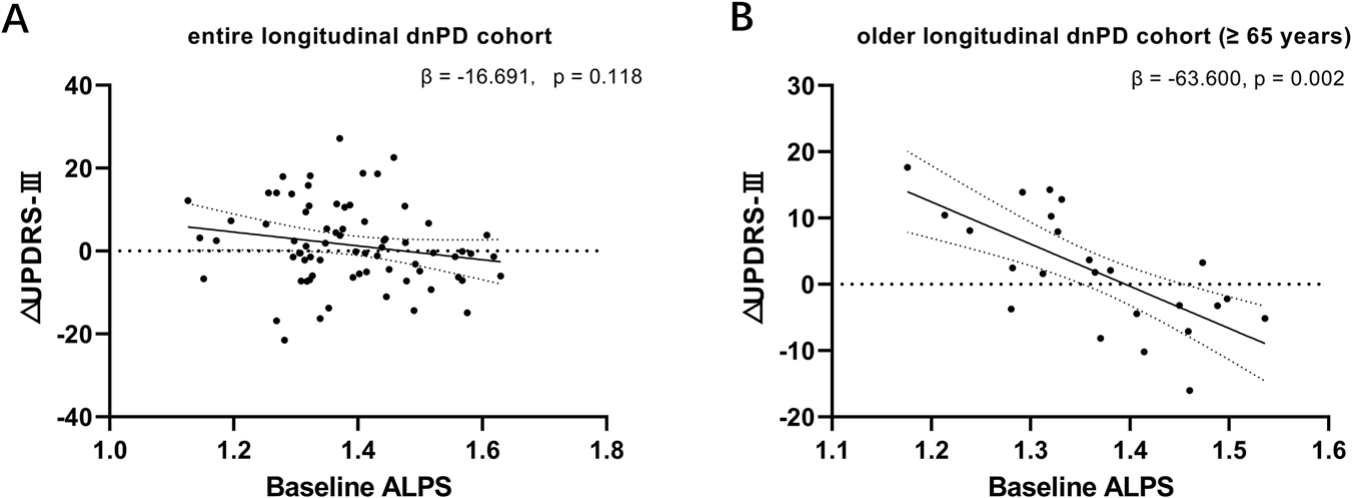
Longitudinal study of dnPD cohort. **A** Correlations of the baseline ALPS index with ΔUPDRS-III score in entire dnPD cohort. **B** Correlations of the baseline ALPS index with ΔUPDRS-III score in the longitudinal cohort of dnPD individuals aged 65 or older. ΔUPDRS-III was defined as the value at the end of follow-up minus the baseline value. Controlling for age, sex, education level, baseline disease duration, follow-up time, LED and baseline UPDRS-III score.

Among all dnPD participants, 72 completed longitudinal follow-up, with an average follow-up duration of 2.5 years (2.49 ± 1.04 years). No significant differences were observed in any demographic or clinical data between the participants completed follow-up and those did not (Table S7). Longitudinal changes in the clinical features and the ALPS index of these subjects are presented in Table 3. Specifically, UPDRS-III showed no increase over time (β = 0.629, SE = 0.250, t = 1.210, p = 0.230), while the H-Y stage exhibited a significant increase (β = 0.074, SE = 0.030, t = 2.447, p = 0.016). Unlike pPD participants, the baseline ALPS index showed no correlation with changes in motor symptoms in the entire dnPD cohort (β = -16.691, 95% CI: −37.726 to 4.343, P = 0.118; Fig. 4A, TABLE S9). However, in dnPD aged 65 or older, a lower baseline ALPS index was associated with a greater longitudinal increase in UPDRS-III (β = -63.600, 95% CI: −99.722 to −27.478, P = 0.002; Fig. 4B, Table S9). At the end of follow-up, 37 dnPD patients received MRI scans. The LMM revealed a statistically significant decline in the ALPS index over time (β = −0.008, SE = 0.004, t = −2.280, p = 0.029, Table 3).

**Table 3:** Time Effects in Linear Mixed-Effects Models for Longitudinal Changes in Clinical Features and the ALPS Index in the dnPD Cohort

	β (SE)	t	P-value
UPDRS-III	0.629(0.520)	1.210	0.230
H-Y stage	0.074(0.030)	2.447	**0.016**
ALPS^a^	−0.008(0.004)	−2.280	**0.029**

Linear mixed-effects models included time, sex, age, baseline disease duration, and LED as fixed effects.

*H-Y* Hoehn and Yahr, *UPDRS-III* Unified Parkinson’s Disease Rating Scale, Part III, *ALPS* diffusion tensor image analysis along the perivascular space.

^a^37 of the 72 participants included in our dnPD cohort received a second MRI scan at the end of follow-up.

Bold values indicate statistical significance (P < 0.05).

## Discussion

The present study investigated glymphatic function in prodromal and early clinical PD. In cross-sectional analyses, we demonstrated that dnPD patients exhibited a reduced ALPS index compared to those in the pPD and HC groups. In pPD aged 65 years or older, the ALPS index was lower than HCs. In longitudinal analyses, patients in the dnPD and pPD groups who progressed to clinical PD exhibited significant decreases in the ALPS index. Furthermore, a lower baseline ALPS index was associated with longitudinal deterioration of motor symptoms. Our results imply that glymphatic dysfunction contributes to PD progression in early stages and may serve as a potential therapeutic target for delaying disease advancement.

The prodromal phase is the earliest stage at which PD clinical research can be conducted. We observed a decreased ALPS index in pPD aged 65 years or older, but not in the middle-to-old age group. This finding aligns with a study that reported a reduced ALPS index in patients with rapid eye movement (REM) sleep behavior disorder (RBD), where the mean patient age was 73 years^14^. Aging is a major risk factor for the decline of glymphatic function^17^. Animal models have demonstrated that, compared to young mice, glymphatic function in elderly mice was reduced by 80%–90%^18^. The same phenomenon was also demonstrated in humans^19^. The driving force of the glymphatic system depends on arterial pulsation, and aging leads to a decrease in vascular compliance. Therefore, age-related reduction in glymphatic function has been suggested to result mainly from reduced interstitial CSF influx due to arterial stiffness. Moreover, it is also associated with diminished CSF production and pressure, impaired aquaporin-4 (AQP4) polarization at the endfeet of astrocytes, and alterations in sleep architecture^5,17^. The interaction effect between age and diagnostic status in our research further demonstrated that glymphatic impairments varied across different age groups. It is noteworthy that the absence of a decline in the ALPS index in middle-to-old pPD participants did not necessarily indicate that glymphatic dysfunction is irrelevant to neurodegeneration in the prodromal phase of PD. Younger patients may have better physiological recovery and higher glymphatic function reserve, allowing them to maintain glymphatic function in the prodromal stages of PD. In contrast, older patients experience age-related reduction in glymphatic function, reducing their capacity for compensation and leading to earlier glymphatic impairment^18^^,^^20^. By including a more representative pPD sample and stratifying participants by age, our study offers a more robust and in-depth understanding of how glymphatic function changes in pPD. Nevertheless, due to the retrospective study design and the lack of dopamine transporter (DAT) scanning, we cannot determine the causal relationships among aging, glymphatic dysfunction, and dopaminergic denervation, nor to clarify whether glymphatic dysfunction is one of the driving factors or merely a concomitant phenomenon of dopaminergic deficits in PD. Future prospective longitudinal cohort studies combining neurotransmitter molecular imaging techniques and ALPS index are expected to elucidate the aforementioned complex relationships.

All dnPD patients recruited for our study were in the early stages of the disease (H-Y stage < 3) and exhibited a reduced ALPS index, consistent with several previous findings^11,12,21^. Notably, Meng et al. and Ma et al. included PD patients already on dopaminergic medication and categorized them into early and late stages based on the H-Y scale. Their studies observed a reduction in the ALPS index only in the late stages, suggesting that the glymphatic system remains unimpaired until later in the disease course^8,9^. However, their study had relatively small sample sizes (29 and 35, respectively) and did not adequately control for confounding factors such as age and sex. In contrast, our study included a larger cohort of early-stage dnPD patients and accounted for potential confounders. Importantly, none of our participants had prior exposure to anti-PD medications. Since oxidative stress and neuroinflammation are common pathological mechanisms in PD^22^ and are linked to glymphatic dysfunction^23^, the use of anti-PD medications with anti-inflammatory and antioxidant properties—such as pramipexole^24^ or rasagiline^25^—could introduce confounding variables when studying glymphatic function. By including a fully drug-naive cohort, we were able to more accurately assess the natural progression of glymphatic function in PD.

Our cross-sectional correlation analysis revealed that age also significantly influenced the association between the ALPS index and clinical characteristics. Specifically, we identified that the correlation between the ALPS index and UPDRS-III scores was significant only in the older dnPD group. This finding was consistent with Cai et al., who reported a negative correlation between the ALPS index and the severity of motor symptoms in PD patients aged 65 years and older, after controlling for disease duration^15^. In addition to motor symptoms, we found that a lower ALPS index was correlated with compromised daily living activities, more non-motor symptoms, poorer sleep quality, and more severe anxiety or depression among older dnPD patients, all impacting their quality of life. Nevertheless, these relationships were not significant in pPD and middle-to-old dnPD groups.

Notably, Yao et al. reported that the ALPS index was positively correlated with ^18^F-fluorodopa striatal standardized uptake value ratio (SUVR) in PD patients^26^, indicating that glymphatic dysfunction may exacerbate the degeneration of dopaminergic neurons. Given that the loss of dopaminergic neurons in the substantia nigra (SN) represents the characteristic pathological hallmark of PD and is strongly associated with its core clinical manifestations—particularly motor symptoms, it is necessary to take the functional status of the dopaminergic system into account when exploring the clinical relevance of the ALPS index. The substantia nigra serves as the primary source of dopaminergic projections to the striatum. PSN-FW reflect the freely diffused water molecules within this region, which may increase due to neuronal degeneration and expansion of the extracellular space. Our previous research demonstrated elevated PSN-FW in pPD and dnPD patients^27^, which was consistent with other multicenter studies^28–31^. Importantly, PSN-FW has been reported to exhibit a negative correlation with dopamine transporter specific binding ratios in the putamen^29–32^. Accordingly, the present study utilized PSN-FW as a surrogate for the integrity of the dopaminergic system and included it as a covariate in correlation analyses. The observed correlations mentioned above remained after controlling for PSN-FW. This suggests that glymphatic dysfunction may independently contribute to clinical symptoms in early-stage PD, potentially through mechanisms distinct from or supplementary to dopaminergic degeneration, such as adverse effects on non-dopaminergic neurons induced by neuroinflammation^33^ or oxidative stress^12^. Further studies incorporating multimodal imaging are warranted to clarify the temporal sequence and mutual interactions among glymphatic dysfunction, dopaminergic degeneration, and clinical manifestations in PD.

Currently, there is limited research on the predictive value of glymphatic function for the progression and phenoconversion of pPD. In our longitudinal analyses, a higher baseline ALPS index was associated with less worsening of both motor and non-motor symptoms in pPD cohort. This may suggest that a preserved glymphatic function may help delay PD progression^3^. Previously, Bae et al. reported that the ALPS index was lower in RBD patients who converted to α-synucleinopathy compared to those who did not, and that the risk of conversion decreased with an increasing ALPS index^14^. The strength of our study lies in the fact that we performed MR scans at both baseline and follow-up. We found that while the ALPS index remained stable in the overall pPD cohort, it decreased by the end of the follow-up period in pPD patients who progressed to a clinical PD diagnosis. This suggested that the conversion from prodromal to clinical PD is accompanied by impairment of the glymphatic system.

The ALPS index could not predict clinical deterioration in the overall dnPD group. However, in dnPD patients aged 65 years or older, we observed a negative correlation between the baseline ALPS index and the ΔUPDRS-III score, suggesting that age may be a significant influencing factor. Si et al. reported no correlation between ΔUPDRS-III score and the baseline ALPS index in a cohort with a mean age of 60.27 years^11^. In contrast, Wood et al. reported a correlation between the baseline ALPS index and the progression of both motor symptoms and cognitive impairments in an older PD cohort (65.3 ± 8.0 y)^13^. Additionally, He et al. also reported that an older PD subgroup (64.1 ± 9.2 y) with a low baseline ALPS index experienced faster deterioration in UPDRS-III score^34^. These findings suggest that the impact of glymphatic dysfunction may be more pronounced in older PD patients.

Given the early impairment of the glymphatic system and its correlation with the severity and progression of PD, the potential of the glymphatic system as a therapeutic target should be emphasized, especially for the older population. Previous research has established the significant influence of sleep on glymphatic function^35,36^, and sleep disorders have been shown to reduce the clearance of α-synuclein in the brain parenchyma of PD patients^37^. This suggests that effective management of sleep disorders may improve glymphatic function and, in turn, benefit PD patients. In addition, cardiorespiratory exercise^38^, omega-3 fatty acid intake^39^, and newly proposed near-infrared light modulation^40^ have been reported to enhance glymphatic function and show promise in reducing the conversion rate from prodromal PD to clinically diagnosed PD, mitigating PD symptoms, and slowing disease progression. Nevertheless, the effectiveness of these innovative treatment strategies requires validation in future studies.

There were several limitations when interpreting the results of the present research. First, both CSF secretion and glymphatic function are regulated by circadian rhythms^41,42^. Although we primarily collected MRI data during the daytime, we have not explored the relationship between the ALPS index and the time of scanning. Second, the ALPS index measures glymphatic activity in a right-to-left direction at the level of the lateral ventricles. Whether it can represent the global glymphatic function requires further corroboration, despite recent research verifying its correlation with classic glymphatic measures^7^. Third, in the pPD and dnPD cohorts, the number of subjects with followed up data was relatively small. To advance the understanding of the causal relationship between glymphatic function and disease progression, particularly in the transition from prodromal phase to clinically diagnosed phase, future research demands larger longitudinal cohort studies and multiple time-point follow-ups. Lastly, although we used PSN-FW as an alternative to DAT imaging, it is crucial to note that PSN-FW may reflect heterogeneous pathology, such as neuroinflammation, rather than solely dopamine neuron loss. The absence of direct DAT imaging limited the definitive dissociation of glymphatic effects from dopaminergic contributions. Future studies integrating DAT imaging with multi-modal MRI are necessary to corroborate these preliminary findings.

In conclusion, we provide strong evidence of early glymphatic dysfunction in PD, as indicated by a reduced ALPS index in early-stage dnPD and older pPD patients. Furthermore, the baseline ALPS index could predict disease progression, particularly in pPD and older dnPD cohorts. Taken together, these results support that glymphatic dysfunction occurs in very early stages and contribute to disease progression.

## Methods

This study was approved by the Ethics Committee of the Affiliated Brain Hospital of Nanjing Medical University. Informed consent was obtained from all participants or their caregivers before enrollment.

### Participants

#### Diagnosis of prodromal PD

A standardized structured questionnaire was used to screen for pPD in the community. Demographic information was routinely collected. Structured interviews were conducted to obtain PD-related risk factors, including occupational solvent/pesticide exposure, coffee/tea use, family history, and non-smoking history. Substantia nigra hyperechogenicity was evaluated by certified ultrasonographers at our institution, with a hyperechoic area ≥ 0.20 cm² defined as abnormal. Mild motor symptoms (MMS) were assessed using the Unified Parkinson’s Disease Rating Scale Part III (UPDRS-III), and scores greater than three points, excluding postural or action tremors, were considered positive. Probable REM sleep behavior disorder (RBD) was identified using the Rapid Eye Movement Sleep Behavior Disorder Questionnaire-Hong Kong (RBDQ-HK), while olfactory dysfunction was measured via the Sniffin’ Sticks test. Orthostatic hypotension (OH) was defined as a decrease in systolic blood pressure of 20 mmHg or more, or a decrease in diastolic blood pressure of 10 mmHg or more, within three minutes of transitioning from a supine to a standing position. Psychiatric symptoms, including depression and anxiety, were evaluated using the Hamilton Depression Scale (HAMD) and Hamilton Anxiety Scale (HAMA), respectively. Non-motor symptoms such as constipation, urinary dysfunction, excessive daytime somnolence, and sexual dysfunction were assessed using the Non-Motor Symptoms Questionnaire for Parkinson’s Disease (PDNMS). Then, according to the Movement Disorder Society (MDS) research criteria for pPD (MDS-pPD)^43^, posterior probability was calculated. Participants with a posterior probability of 80% or higher were diagnosed with pPD.

#### Diagnosis of clinical PD

Patients with de novo PD (dnPD) were recruited from the Neurology Department of the Affiliated Brain Hospital of Nanjing Medical University between September 2017 and December 2023. All patients met the following criteria: (1) newly diagnosed with PD based on the United Kingdom Parkinson’s Disease Society Brain Bank clinical diagnostic criteria^44^; (2) currently untreated; (3) early stage (modified Hoehn and Yahr scores [H-Y stages] <3); (4) follow-up through the hospital for at least 1 year to confirm the PD diagnosis; and (5) a positive response to levodopa (L-dopa) therapy in subsequent treatment.

The exclusion criteria were as follows: (1) atypical or secondary Parkinsonism; (2) clinically significant lesions found by brain MRI; (3) severe physical illness or clinically confirmed psychiatric disorders; and (4) difficulty in cooperating with clinical assessments.

Health controls (HCs) were recruited from the community. They reported no symptoms associated with PD and were clinically evaluated by two experienced neurologists. According to the World Health Organization (WHO), the age of 65 is recommended as the cut off value for the elderly, combined with the research which reported a sharp decrease in the glymphatic function in patients aged 65 years or older^16^, each group was divided into 2 subgroups, namely, middle-to-old pPD group (pPDm), older pPD group (pPDo), middle-to-old dnPD group (dnPDm), older dnPD group (dnPDo), middle-to-old HC group (HCm), and older HC group (HCo).

This study utilized a retrospective cohort design incorporating longitudinal follow-up. All follow-up data were collected until the point of data analysis for this study. Specifically, for pPD participants, follow-up was scheduled to be conducted approximately 1.5 years after each participant’s initial enrollment, at which time they were asked to complete a second comprehensive assessment and MR scan based on their willingness. In addition, some participants proactively visited the neurology clinic due to the onset of PD-related clinical symptoms or the worsening of their existing MMS, at which point a comprehensive clinical assessment and MR scan were also performed. For dnPD patients, follow-up assessments and MR scans were scheduled to be conducted approximately 2–3 years after initial enrollment, or when patients returned to our neurology clinic for further PD-related management. This protocol permitted both scheduled and event-driven clinical follow-up, thereby more closely reflecting real-world clinical practice.

### Clinical and neuropsychological assessments

The severity of PD was assessed using modified H-Y stages. The motor symptoms and daily living activities were evaluated using the UPDRS III and II. We assessed non-motor symptoms and sleep quality using the PDNMS and the Parkinson’s Disease Sleep Scale (PDSS), respectively. To assess global cognitive performance, we used the Montreal Cognitive Assessment (MoCA). HAMD and HAMA were used to evaluated the psychiatric symptoms. The current L-dopa equivalent daily dose (LED) was calculated during follow-up based on a previously described method^45^.

### Magnetic resonance imaging acquisition

All participants were scanned with a 3-T Verio Siemens scanner (Siemens, Verio, Munich, Germany) in a supine position with their heads secured using foam pads to reduce movement. The DTI data were obtained using a spin-echo echo-planar imaging sequence in axial planes parallel to the anterior-posterior commissure line. The parameters were as follows: TR = 9000 ms, TE = 104 ms, flip angle = 7°, FOV = 230 × 230 mm, thickness = 2.5 mm with no gap between slices, matrix = 128 × 128, 49 slices covered the global brain, b-values = 0 and 1000 s/mm², and 64 diffusion gradient directions.

### ALPS index calculation

Raw diffusion data were first preprocessed using MRtrix3 (http://www.mrtrix.org) and FMRIB’s Software Library (FSL, https://fsl.fmrib.ox.ac.uk/fsl/fslwiki/FSL/), including signal denoising, Gibbs ringing removal, eddy-current and head motion correction, bias field correction, and brain masking.

DTI ALPS index were calculated using an validated method^46^. This automated pipeline demonstrated favorable inter-scanner reproducibility, inter-rater reliability, and test-retest repeatability. Briefly, the FA maps and diffusivities maps [Dxx, Dyy, Dzz] were acquired from preprocessed diffusion data using dtifit and then co-registered to the FA map template (JHU-ICBM-FA-1 mm). Four spherical ROIs with 5 mm-diameter were automatically defined in the areas of bilateral projection fiber (proj) and association fiber (assoc) which were applied on the diffusivity maps of all subjects. The center coordinates of ROIs were as follows: left projection (116,110,99), left association (128,110,99), right projection (64,110,99) and right association (51,110,99) on the JHU-ICBM-FA template. The left and right ALPS index was calculated as [(Dxx-proj + Dxx-assoc) / (Dyy-proj + Dzz-assoc)] on each side, respectively. The mean ALPS index is defined by the average of bilateral ALPS indexes.

### Free water analysis

The calculation of PSN-FW was consistent with our prior research^27^. Briefly, after the preprocessing steps, the free water maps were estimated using the script provided by the MarkVCID projects (https://markvcid.partners.org/markvcid1-protocols-resources). Subsequently, the b0 images were normalized to the Montreal Neurological Institute (MNI) space via linear registration, and the free water maps were transformed into the MNI space using the derived transformation matrices. Bilateral PSN ROIs were hand-drawn on the b0 images to extract free water values. Finally, the free water values obtained from bilateral ROIs were averaged to derive the PSN-FW.

### Statistical analysis

Data were analyzed using SPSS Statistics version 26.0 (IBM Corporation) and R (version 4.2.3). The results were expressed as the mean ± standard deviation (SD) for continuous variables and as frequencies for categorical variables. The Kolmogorov-Smirnov test was performed to assess the normality of the distribution of continuous variables. Two-sample t-tests, Mann-Whitney U test, chi-square tests, Kruskal–Wallis W test, and analysis of variance (ANOVA) were conducted to examine inter-group differences in demographic and clinical data.

For cross-sectional data, the analysis of covariance (ANCOVA) was utilized to investigate inter-group differences in the ALPS index, with age, sex, and education level as covariates. Bonferroni correction was used for post hoc tests. Partial correlation was used to explore the relationship between the ALPS index and clinical features at the baseline, controlling for age, sex, and education level. For dnPD group, disease duration was controlled as an additional covariate. To further investigate whether the association between the ALPS index and the severity of clinical symptoms is independent of dopaminergic system integrity, we conducted supplementary partial correlation analyses with PSN-FW included as an additional covariate. Multiple comparisons for correlation analyses were corrected using false discovery rate (FDR) correction (Benjamini-Hochberg), with an adjusted P < 0.05.

For longitudinal analyses, changes in clinical features and the ALPS index over time were analyzed using linear mixed-effects models (LMMs, lme4 R packages). The model used each clinical feature and the ALPS index at all time points as the dependent variable, with follow-up time, sex, and age included as fixed effects. For the dnPD cohort, baseline disease duration and LED were additionally included as fixed effects. The effect of follow-up time represented the average annualized change in each clinical feature and the ALPS index. The relationship between the baseline ALPS index and the change in clinical features was evaluated using multivariable linear regression analysis, controlling for age, sex, education level, and the corresponding baseline clinical feature in the longitudinal pPD cohort. In the longitudinal dnPD cohort, baseline disease duration, LED, and follow-up time were controlled as additional covariates.

## Supplementary information

Revised Supplementary Materials

## Data Availability

The original data for this study can be obtained from the corresponding author via email upon reasonable request.
