# Supplementary material for: Perivascular space fluid diffusivity predicts clinical deterioration in prodromal and early-stage Parkinson’s disease
Source: NPJ Parkinsons Dis. 2025 Jun 14;11:169. doi: 10.1038/s41531-025-01036-6 (PMC12167374; doi:10.1038/s41531-025-01036-6)
Supplement: Supplementary file 1 — Revised Supplementary Materials [file 41531_2025_1036_MOESM1_ESM.docx]

**TABLE S1 Inter-group Comparison of the ALPS index**

|  | HC | pPD | dnPD |  | post-hoc | | |
| --- | --- | --- | --- | --- | --- | --- | --- |
|  |  |  |  | P | P* | P** | P*** |
| **All participants** | | | | | | | |
| ALPS index | 1.434±0.119 | 1.396±0.152 | 1.359±0.140 | **0.001** | 0.856 | **0.001** | **0.040** |
| **Participants < 65 years old** | | | | | | | |
| ALPS index | 1.433±0.124 | 1.458±0.129 | 1.381±0.133 | **0.002** | 1.000 | **0.027** | **0.004** |
| **Participants ≥ 65 years old** | | | | | | | |
| ALPS index | 1.438±0.069 | 1.306±0.139 | 1.314±0.143 | **0.028** | **0.044** | **0.027** | 1.000 |

ANCOVA was used to examine group differences, controlling for age, sex, and education level. Bonferroni correction was applied for post hoc tests.

P*: HC vs pPD, P**: HC vs dnPD, P***: pPD vs dnPD

**TABLE S2 Demographic and Clinical Data of Participants Aged Less Than 65**

|  |  | HC | pPD | dnPD |  | Post-hoc | | |
| --- | --- | --- | --- | --- | --- | --- | --- | --- |
|  |  |  |  |  | P | P* | P** | P*** |
| Age（years） |  | 56.74±4.37 | 54.43±7.16 | 55.00±6.33 | 0.055^a^ | - | - | - |
| Sex (Male %) |  | 46.15 | 50.00 | 54.47 | 0.510^b^ | - | - | - |
| Disease duration (years) |  | - | - | 1.83±1.48 | **-** | - | **-** | - |
| Education（years） |  | 10.82±3.76 | 10.96±4.22 | 9.68±4.24 | **0.018**^c^ | 1.000 | **0.033** | **0.026** |
| H-Y stage |  | - | - | 2(1, 2) | **-** | - | - | - |
| UPDRS-Ⅱ |  | - | - | 7.68±3.56 | **-** | - | - | - |
| UPDRS-Ⅲ |  | - | 8.55±3.02 | 23.76±8.89 | **<0.001**^d^ | - | - | - |
| PDSS |  | - | - | 127.55±22.72 | - | - | - | - |
| PDNMS |  | - | 7.77±3.37 | 7.59±4.43 | 0.525^d^ | - |  | - |
| MoCA |  | 26.89±1.35 | 23.33±4.32 | 22.18±4.63 | **<0.001**^c^ | **<0.001** | **<0.001** | 0.157 |
| HAMA |  | - | 6.83±4.09 | 6.67±5.67 | 0.334^d^ | **-** | **-** | - |
| HAMD |  | - | 8.11±5.01 | 9.32±7.00 | 0.609^d^ | **-** | **-** | - |

^a^Analysis of Variance.

^b^Chi-square tests.

^c^Kruskal–Wallis W test.

^d^Mann-Whitney U test.

P*: HC vs pPD, P**: HC vs dnPD, P***: pPD vs dnPD.

Abbreviations: HC, healthy control; pPD, prodromal Parkinson’s disease; dnPD, de novo Parkinson’s disease; H-Y, Hoehn and Yahr; UPDRS-Ⅱ, Unified Parkinson’s Disease Rating Scale, Part Ⅱ; UPDRS-III, Unified Parkinson’s Disease Rating Scale, Part III; PDSS, Parkinson’s Disease Sleep Scale; PDNMS, Parkinson; disease Non-Motor Symptoms Questionnaire; MoCA, Montreal Cognitive Assessment; HAMD, Hamilton Depression Rating Scale; HAMA, Hamilton Anxiety Scale.

**TABLE S3 Demographic and Clinical Data of Participants Aged 65 or Over**

|  |  | HC | pPD | dnPD |  | Post-hoc | | |
| --- | --- | --- | --- | --- | --- | --- | --- | --- |
|  |  |  |  |  | P | P* | P** | P*** |
| Age（years） |  | 66.64±2.20 | 68.57±3.04 | 68.52±2.65 | 0.099^a^ | - | - | - |
| Sex (Male %) |  | 54.54 | 56.76 | 38.33 | 0.177^b^ | - | - | - |
| Disease duration (years) |  | - | - | 2.28±3.55 | **-** | - | **-** | - |
| Education（years） |  | 10.73±5.12 | 9.43±4.35 | 8.23±4.79 | 0.146^c^ | - | **-** | **-** |
| H-Y stage |  | - | - | 2(1.5, 2) | **-** | - | - | - |
| UPDRS-Ⅱ |  | - | - | 7.92±3.90 | **-** | - | - | - |
| UPDRS-Ⅲ |  | - | 9.11±5.30 | 24.82±8.46 | **<0.001**^d^ | - | - | - |
| PDSS |  | - | - | 121.48±26.33 | - | - | - | - |
| PDNMS |  | - | 9.57±6.04 | 7.88±4.16 | 0.287^d^ | - |  | - |
| MoCA |  | 26.00±2.24 | 22.05±4.73 | 20.07±6.15 | **0.029**^c^ | 0.093 | **0.019** | 0.281 |
| HAMA |  | - | 6.76±6.04 | 6.75±4.88 | 0.610^d^ | **-** | **-** | - |
| HAMD |  | - | 8.35±6.97 | 9.52±6.54 | 0.274^d^ | **-** | **-** | - |

^a^Analysis of Variance.

^b^Chi-square tests.

^c^Kruskal–Wallis W test.

^d^Mann-Whitney U test.

P*: HC vs pPD, P**: HC vs dnPD, P***: pPD vs dnPD.

Abbreviations: HC, healthy control; pPD, prodromal Parkinson’s disease; dnPD, de novo Parkinson’s disease; H-Y, Hoehn and Yahr; UPDRS-Ⅱ, Unified Parkinson’s Disease Rating Scale, Part Ⅱ; UPDRS-III, Unified Parkinson’s Disease Rating Scale, Part III; PDSS, Parkinson’s Disease Sleep Scale; PDNMS, Parkinson; disease Non-Motor Symptoms Questionnaire; MoCA, Montreal Cognitive Assessment; HAMD, Hamilton Depression Rating Scale; HAMA, Hamilton Anxiety Scale.

**TABLE S4 Partial correlation of ALPS Index and Clinical Features at the baseline**

|  | All  participants | |  | Participants < 65 years old | |  | Participants ≥ 65 years old | |  |
| --- | --- | --- | --- | --- | --- | --- | --- | --- | --- |
|  | r | p | FDR-p | r | p | FDR-p | r | p | FDR-p |
| Prodromal PD cohort | | | | | | | | | |
| UPDRS-Ⅲ | -0.119 | 0.271 | 0.407 | -0.010 | 0.943 | 0.943 | -0.209 | 0.235 | 0.705 |
| PDNMS | 0.031 | 0.775 | 0.775 | 0.085 | 0.556 | 0.834 | -0.018 | 0.920 | 0.995 |
| MoCA | 0.185 | 0.084 | 0.252 | 0.427 | **0.002** | **0.006** | 0.001 | 0.995 | 0.995 |
| De novo PD cohort | | | | | | | | | |
| UPDRS-Ⅱ | -0.100 | 0.182 | 0.182 | 0.037 | 0.686 | 0.784 | -0.309 | **0.020** | **0.032** |
| UPDRS-Ⅲ | -0.134 | 0.074 | 0.148 | -0.096 | 0.300 | 0.728 | -0.299 | **0.025** | **0.033** |
| H-Y | -0.186 | **0.012** | **0.048** | -0.209 | **0.022** | 0.176 | -0.102 | 0.456 | 0.456 |
| PDSS | 0.184 | **0.014** | **0.048** | 0.069 | 0.455 | 0.728 | 0.433 | **<0.001** | **0.007** |
| PDNMS | -0.176 | **0.018** | **0.048** | -0.114 | 0.217 | 0.728 | -0.369 | **0.005** | **0.013** |
| MoCA | 0.121 | 0.106 | 0.170 | 0.070 | 0.451 | 0.728 | 0.264 | **0.049** | 0.056 |
| HAMA | -0.112 | 0.134 | 0.178 | -0.041 | 0.654 | 0.784 | -0.314 | **0.019** | **0.032** |
| HAMD | -0.106 | 0.156 | 0.178 | 0.012 | 0.895 | 0.895 | -0.375 | **0.004** | **0.013** |

All correlation analyzes above were adjusted for age, sex, education level and disease duration. Multiple comparisons for correlation analyses were corrected using false discovery rate (FDR) correction (Benjamini-Hochberg), with an adjusted P < 0.05. Abbreviations: H-Y, Hoehn and Yahr; UPDRS-Ⅱ, Unified Parkinson’s Disease Rating Scale, Part Ⅱ; UPDRS-III, Unified Parkinson’s Disease Rating Scale, Part III; PDSS, Parkinson’s Disease Sleep Scale; PDNMS, Parkinson; disease Non-Motor Symptoms Questionnaire; MoCA, Montreal Cognitive Assessment; HAMD, Hamilton Depression Rating Scale; HAMA, Hamilton Anxiety Scale.

**TABLE S5 Partial correlation of ALPS Index and Clinical Features at the baseline (Corrected for FW in PSN)**

|  | All  participants | |  | Participants < 65 years old | |  | Participants ≥ 65 years old | |  |
| --- | --- | --- | --- | --- | --- | --- | --- | --- | --- |
|  | r | p | FDR-p | r | p | FDR-p | r | p | FDR-p |
| Prodromal PD cohort | | | | | | | | | |
| UPDRS-Ⅲ | -0.153 | 0.161 | 0.242 | -0.057 | 0.695 | 0.695 | -0.222 | 0.215 | 0.645 |
| PDNMS | -0.014 | 0.899 | 0.899 | 0.058 | 0.690 | 0.695 | -0.071 | 0.696 | 0.908 |
| MoCA | 0.210 | 0.051 | 0.153 | 0.463 | **0.001** | **0.003** | 0.021 | 0.908 | 0.908 |
| De novo PD cohort | | | | | | | | | |
| UPDRS-Ⅱ | -0.096 | 0.201 | 0.201 | 0.044 | 0.633 | 0.791 | -0.305 | **0.023** | **0.037** |
| UPDRS-Ⅲ | -0.129 | 0.085 | 0.170 | -0.090 | 0.331 | 0.774 | -0.297 | **0.028** | **0.037** |
| H-Y | -0.179 | **0.017** | 0.056 | -0.200 | **0.030** | 0.240 | -0.096 | 0.485 | 0.485 |
| PDSS | 0.181 | **0.016** | 0.056 | 0.066 | 0.478 | 0.774 | 0.430 | **0.001** | **0.008** |
| PDNMS | -0.172 | **0.021** | 0.056 | -0.109 | 0.238 | 0.774 | -0.372 | **0.005** | **0.016** |
| MoCA | 0.120 | 0.110 | 0.176 | 0.065 | 0.484 | 0.774 | 0.269 | **0.047** | 0.053 |
| HAMA | -0.106 | 0.158 | 0.201 | -0.037 | 0.692 | 0.791 | -0.306 | **0.023** | **0.036** |
| HAMD | -0.102 | 0.176 | 0.201 | 0.015 | 0.870 | 0.870 | -0.369 | **0.006** | **0.016** |

All correlation analyzes above were adjusted for age, sex, education level disease duration and FW in PSN. Multiple comparisons for correlation analyses were corrected using false discovery rate (FDR) correction (Benjamini-Hochberg), with an adjusted P < 0.05. Abbreviations: H-Y, Hoehn and Yahr; UPDRS-Ⅱ, Unified Parkinson’s Disease Rating Scale, Part Ⅱ; UPDRS-III, Unified Parkinson’s Disease Rating Scale, Part III; PDSS, Parkinson’s Disease Sleep Scale; PDNMS, Parkinson; disease Non-Motor Symptoms Questionnaire; MoCA, Montreal Cognitive Assessment; HAMD, Hamilton Depression Rating Scale; HAMA, Hamilton Anxiety Scale.

**TABLE S6 Comparison of Demographic and Clinical Data Between pPD Participants With and Without Follow-Up**

|  | Without Follow-Up | With Follow-Up | χ2/t/z | p |
| --- | --- | --- | --- | --- |
| Age（years） | 57.98±9.24 | 64.86±6.82 | -3.576 | **0.001^a^** |
| Sex (Male %) | 51.61 | 55.17 | 0.100 | 0.751^b^ |
| Education（years） | 10.15±4.91 | 10.76±2.68 | -0.060 | 0.952^c^ |
| UPDRS-Ⅲ | 9.02±3.26 | 8.28±5.48 | -1.460 | 0.144^c^ |
| PDNMS | 8.02±5.02 | 9.55±4.43 | -1.614 | 0.106^c^ |
| MoCA | 22.73±5.20 | 23.00±2.54 | -0.829 | 0.407^c^ |
| HAMA | 6.51±5.08 | 7.41±4.70 | -1.104 | 0.270^c^ |
| HAMD | 7.75±5.78 | 9.17±6.02 | -0.948 | 0.343^c^ |

^a^Independent-sample t test.

^b^Chi-square tests.

^c^Mann-Whitney U test.

Abbreviations: pPD, prodromal Parkinson’s disease; UPDRS-III, Unified Parkinson’s Disease Rating Scale, Part III; PDNMS, Parkinson; disease Non-Motor Symptoms Questionnaire; MoCA, Montreal Cognitive Assessment; HAMD, Hamilton Depression Rating Scale; HAMA, Hamilton Anxiety Scale.

**TABLE S7 Comparison of Demographic and Clinical Data Between dnPD Participants With and Without Follow-Up**

|  | Without Follow-Up | With Follow-Up | χ2/t/z | p |
| --- | --- | --- | --- | --- |
| Age（years） | 60.23±8.28 | 58.19±8.33 | 1.623 | 0.106^a^ |
| Sex (Male %) | 48.65 | 50.00 | 0.032 | 0.858^b^ |
| Disease duration (years) | 2.10±2.88 | 1.78±1.17 | -0.843 | 0.399^c^ |
| Education（years） | 8.92±4.59 | 9.64±4.26 | -0.522 | 0.602^c^ |
| H-Y stage | 1.5(2, 2) | 1.5(2, 2) | -0.944 | 0.345^c^ |
| UPDRS-Ⅱ | 7.87±3.75 | 7.58±3.56 | -0.621 | 0.535^c^ |
| UPDRS-Ⅲ | 24.40±7.93 | 23.67±8.64 | -0.816 | 0.415^c^ |
| PDSS | 124.55±24.40 | 127.07±23.64 | -1.036 | 0.300^c^ |
| PDNMS | 7.79±4.27 | 7.53±4.45 | -0.534 | 0.593^c^ |
| MoCA | 21.11±5.70 | 22.07±4.48 | -0.758 | 0.449^c^ |
| HAMA | 6.67±4.95 | 6.75±6.09 | -0.529 | 0.597^c^ |
| HAMD | 9.47±6.91 | 9.25±6.77 | -0.392 | 0.695^c^ |

^a^Independent-sample t test..

^b^Chi-square tests.

^c^Mann-Whitney U test.

Abbreviations: dnPD, de novo Parkinson’s disease; H-Y, Hoehn and Yahr; UPDRS-Ⅱ, Unified Parkinson’s Disease Rating Scale, Part Ⅱ; UPDRS-III, Unified Parkinson’s Disease Rating Scale, Part III; PDSS, Parkinson’s Disease Sleep Scale; PDNMS, Parkinson; disease Non-Motor Symptoms Questionnaire; MoCA, Montreal Cognitive Assessment; HAMD, Hamilton Depression Rating Scale; HAMA, Hamilton Anxiety Scale.

**TABLE S8 Multivariable linear regression analysis of the baseline ALPS index and the progression of motor and non-motor symptoms in longitudinal pPD cohort**

| Variable | Coefficient (95% CI) | Standard coefficient | t | p |
| --- | --- | --- | --- | --- |
| Model 1: Change in UPDRS-Ⅲ score as dependent variable | | | | |
| ALPS_BL | -21.418 (-42.570, -0.266) | -0.504 | -2.095 | **0.047** |
| UPDRS-Ⅲ_BL | -0.060 (-0.600, 0.480) | -0.046 | -0.231 | 0.819 |
| Sex | -0.753 (-0.658, 5.151) | -0.053 | -0.264 | 0.794 |
| Age | -0.646 (-1.184, -0.108) | -0.612 | -2.483 | **0.021** |
| Education | -0.712 (-1.761, 0.338) | -0.265 | -1.402 | 0.174 |
| Model 2: Change in PDNMS score as dependent variable | | | | |
| ALPS_BL | -9.958 (-18.310, -1.606) | -0.496 | -2.466 | **0.022** |
| PDNMS_BL | -0.322 (-0.589, -0.055) | -0.420 | -2.492 | **0.020** |
| Sex | -1.011 (-3.373, 1.350) | -0.151 | -0.886 | 0.385 |
| Age | -0.185 (-0.402, 0.032) | -0.372 | -1.766 | 0.091 |
| Education | 0.192 (-0.224, 0.608) | 0.152 | 0.955 | 0.350 |

In model 1, the dependent variable was the change in UPDRS-Ⅲ score (ΔUPDRS-Ⅲ). In model 2, the dependent variable was the change in PNNMS score (ΔPDNMS). ΔUPDRS-Ⅲ and ΔPDNMS were defined as the value at the end of follow-up minus the baseline value.

Abbreviation: ALPS, diffusion tensor image analysis along the perivascular space; pPD, prodromal Parkinson’s disease; BL, baseline; CI, confidence interval; UPDRS-III, Unified Parkinson’s Disease Rating Scale, Part III; PDNMS, Parkinson disease Non-Motor Symptoms Questionnaire

**TABLE S9 Multivariable linear regression analysis of the baseline ALPS index and the progression of motor symptoms in longitudinal dnPD cohort**

| Variable | Coefficient (95% CI) | Standard coefficient | t | p |
| --- | --- | --- | --- | --- |
| Model 1: In the entire longitudinal dnPD cohort | | | | |
| ALPS_BL | -16.691 (-37.726, 4.343) | -0.173 | -1.586 | 0.118 |
| UPDRS-Ⅲ_BL | -0.671 (-0.968, -0.374) | -0.476 | -4.512 | **<0.001** |
| Sex | -5.705 (-11.259, -0.151) | -0.236 | -2.053 | **0.044** |
| Age | 0.034 (-0.283, 0.351) | 0.023 | 0.214 | 0.831 |
| Education | -0.657 (-1.282, -0.031) | -0.226 | -2.099 | **0.040** |
| Disease duration_BL | -0.628 (-2.761, 1.505) | -0.061 | -0.589 | 0.558 |
| Follow-up time | 0.981 (-1.961, 3.923) | 0.083 | 0.667 | 0.508 |
| LED | -0.001 (-0.012, 0.010) | -0.023 | -0.195 | 0.846 |
| Model 2: In the longitudinal cohort of dnPD individuals aged 65 or older | | | | |
| ALPS_BL | -63.600 (-99.722, -27.478) | -0.727 | -3.753 | **0.002** |
| UPDRS-Ⅲ_BL | -0.846 (-1.245, -0.447) | -0.810 | -4.520 | **<0.001** |
| Sex | -6.172 (-17.141, 4.797) | -0.270 | -1.199 | 0.249 |
| Age | 0.806 (-0.546, 2.159) | 0.233 | 1.271 | 0.223 |
| Education | -1.327 (-2.417, -0.237) | -0.519 | -2.594 | **0.020** |
| Disease duration_BL | -2.268 (-5.659, 1.123) | -0.262 | -1.426 | 0.174 |
| Follow-up time | -0.631 (-5.136, 3.874) | -0.057 | -0.298 | 0.769 |
| LED | 0.005 (-0.015, 0.025) | 0.096 | 0.555 | 0.587 |

Model 1 included all participants in our longitudinal dnPD cohort. Model 2 included participants aged 65 years or older at baseline in our longitudinal dnPD cohort. ΔUPDRS-Ⅲ were defined as the value at the end of follow-up minus the baseline value.

Abbreviation: ALPS, diffusion tensor image analysis along the perivascular space; dnPD, de novo Parkinson’s disease; BL, baseline; CI, confidence interval; UPDRS-III, Unified Parkinson’s Disease Rating Scale, Part III; LED, L-dopa equivalent daily dose.
